# Is Specific Learning Disorder Predicted by Developmental Language Disorder? Evidence from a Follow-Up Study on Italian Children

**DOI:** 10.3390/brainsci13040701

**Published:** 2023-04-21

**Authors:** Pasquale Rinaldi, Arianna Bello, Ilaria Simonelli, Maria Cristina Caselli

**Affiliations:** 1 Institute of Cognitive Sciences and Technologies, National Research Council, Via Nomentana 56, 00161 Rome, Italy; cristina.caselli@istc.cnr.it; 2 Department of Education, Roma Tre University, Via Castro Pretorio 20, 00185 Rome, Italy; arianna.bello@uniroma3.it; 3 Clinical Trial Center Fatebenefratelli Tiberina Island–Gemelli Island, Via di Ponte Quattro Capi 39, 00186 Rome, Italy; ilaria.simonelli@afar.it

**Keywords:** predictors of specific learning disorders, continuity from DLD and SLD, proximal and distal predictors of SLD

## Abstract

Specific Learning Disorder (SLD) is a complex disorder with a strong genetic component, characterized by varying manifestations and considerable differences among children. Several studies have highlighted that difficulties in language acquisition and the presence of Developmental Language Disorders (DLDs) are frequently associated with SLD, suggesting a continuity between the two disorders. This study aimed to add evidence on the proximal and distal predictors of SLD, focusing on the eventual continuity for the presence of DLD at 4–5 years, on some linguistic and communicative abilities at 27–30 months, and on biological and environmental factors. Our sample consisted of 528 families, whose children (Italian monolingual) participated in a screening program at the age of 27–30 months. When children were on average 8.05 years old, parents were asked to answer an interview aimed at collecting information about the children’s language and learning development. Results showed that the prevalence of children with an SLD (7.01%) was in line with those reported in other similar studies. The diagnosis of SLD was significantly predicted by the previous diagnosis of DLD, by male sex/gender, and by the familial risk of SLD. Children with these characteristics had a 54% probability of presenting an SLD.

## 1. Introduction

The current study aims to investigate eventual predictors of Specific Learning Disorder (SLD), focusing on biological and environmental factors, as well as on the linguistic and communicative abilities of children of preschool age, particularly the presence of Developmental Language Disorder (DLD) at 4–5 years of age and/or the presence of Language Delay (LD) at 27–30 months of age.

SLD is a complex disorder with varying manifestations and considerable differences in interpersonal characteristics; it is present worldwide. SLD is a general term referring to a group of disorders that may involve difficulties in reading (dyslexia), written expression (dysgraphia), and/or mathematics (dyscalculia); however, it is not accounted for by low intelligence (IQ), sensory acuity (visual problems), poor learning opportunities, or intellectual disabilities [1,2]. In Italy, three consensus conferences on SLD have been celebrated, the last one in 2021, whose guidelines were published 20 January 2022 [3]. SLD is readily apparent in the early school years in most individuals; symptoms are usually detected when students show a learning profile that is qualitatively lower than their chronological and mental age.

### 1.1. Epidemiology, Biological, and Environmental Risk Factors

The prevalence of SLD varies between 3% and 12% among the general population, depending on factors such as cut-offs used for identification [4,5], sex/gender (boy:girl ratio 2–3.7:1) [6] (but see also [5]), age of assessment, assessment tools [7,8], and sample size [5]. Between 2008 and 2013, a study on a sample of nearly 10,000 Italian 8- to 10-year-old primary school students found that only 1.3% of them had already received a diagnosis of SLD, despite the fact that 3.5% of the sample showed dyslexia according to the diagnostic tools used in the study [9].

Several studies highlighted a genetic component; for example, heritability estimates from family and twin studies varied between 40% and 70% [10,11]. By reviewing the literature from the past 20 years, Georgitsi and colleagues underscored that the genetic architecture of SLD is not specific and that SLD, particularly dyslexia, is marked by high-genetic heterogeneity [12].

Another open question is why SLD seems to be more prevalent worldwide in boys than in girls. Arnett and colleagues suggested that it could be partially explained by cognitive correlates emerging prior to schooling, such as reading ability (slower processing speed in boys), which could serve as a proxy for the sex difference in brain development [13]. From the biological perspective, however, convincing genetic evidence to explain the sex bias observed in SLD is still lacking or is at least contradictory. See also [14,15] for a discussion on sex/gender differences in children with typical and atypical language development.

Although there is agreement on the genetic contributions to SLD appearing to be unquestionable, the contribution of environmental factors is still being debated. Several studies have highlighted that genetic and environmental factors could act in a combined way. For example, a family history of reading difficulties and parental literacy skills has been found to predict literacy problems or SLD in offspring [16,17]. In addition, parental education, particularly maternal education level, and parental reading habits were found to be significantly associated with children’s reading ability, suggesting that mothers may create different reading environments for their children that could positively influence children’s reading acquisition, contrasting biological risk factors. The disadvantages of having less-educated parents could be related to parental suboptimal reading abilities and to the educative practices adopted. See Ref. [18] for a systematic review on this topic.

### 1.2. Proximal and Distal Predictors of SLD and DLD

Several studies suggested a continuity from early LD to DLD to SLD (see Ref. [19] among others). Indeed, difficulties in language acquisition and the presence of DLDs (in particular) frequently occurred in children with SLD [20,21]. Furthermore, several Italian studies reported a high incidence of language impairment in the preschool years in children with SLD, indicating the presence of a moderate but widespread linguistic deficit [22,23].

Language and reading are both viewed as highly heritable traits that are likely to share common genetic and/or neurobiological influences [24]. Poor language skills and/or the presence of DLD as risk factors of SLD have also been found in several studies [20,25,26]. The comorbidity rate between DLD and dyslexia has also been found to be higher than expected (in the order of 50%) on the basis of independent single deficits [24].

Children with DLD and SLD could have phonological deficits or could have intact phonological skills [27]. However, some children with DLD have no difficulties in reading [28,29]. In this regard, several studies have argued that SLD and DLD are distinct disorders [28,30,31,32] and show different developmental trajectories [27]. Some children recover their language difficulties, whereas others continue to present phonological weaknesses associated with serious delay in vocabulary and grammar. In children with dyslexia, specific deficits in phonological aspects of language are frequently observed in preschoolers, without similar difficulties in the broader language domain [33,34].

Looking at distal risk factors or early precursors of SLD, a longitudinal study by van Viersen and colleagues found that children with SLD with a familial risk of SLD had a lower vocabulary size and lower initial growth rates of receptive and productive vocabulary size from 17 to 35 months of age compared with those with SLD without a familial risk of SLD, as well as those with typical development. The authors suggested that the early vocabulary growth of children with SLD and familial risk of SLD is characterized by a delay but not by a deviance of growth; thus, vocabulary should be considered a risk factor of SLD [35]. A subsequent study on the same sample of children showed that among the vocabulary measures, the most sensitive predictors of later reading development appeared to be the vocabulary size and the proportion of verbs at 23 months of age, as well as the proportion of closed class words up to 35 months of age [36], partially confirming the results that emerged from Ref. [37]. Several studies (e.g., [21,38,39]) found an association between early LD (children identified as late talkers) and low outcomes in reading and spelling throughout the school years that became more evident as literacy demands increased over time.

Furthermore, the continuity between early LD and DLD is still an open question. Some children with LD progress to DLD, whereas some do not. In a predictive study, Desmarais and colleagues failed to find linguistic measures collected when children were 2 years old that were able to predict DLD at 4 years of age [40]. However, Hsu and colleagues found that vocabulary and gesture production at 15 months of age contributed to later DLD risk [41]. In her meta-analysis, Fisher highlighted that receptive and expressive lexical skills could explain a small (but significant) amount of the variance in the outcome of children with LD [42]. In a subsequent longitudinal study, Chilosi and colleagues confirmed that the severity of receptive and expressive lexical and grammar delays predicted the diagnosis of DLD among children with LD [43].

Children with early LD frequently show weaknesses of various degrees of severity in several communicative and linguistic aspects (other than receptive and expressive vocabulary). For example, Bello and colleagues showed that a great number of children with LD also had weaknesses in gesture production [44], decontextualized comprehension, and verbal imitation, confirming the results of previous studies that used both parent reports and direct observation [45,46,47]. However, studies that have specifically investigated the use of gestures in children with LD as a predictive factor have provided conflicting findings [48]. A recent Italian study on children with LD failed to identify vocabulary size and gesture production at 3 years of age as significant measures useful in distinguishing children who later developed a DLD from those who did not [49].

A lot of studies, not mentioned above, identified a strong relationship between linguistic and cognitive abilities of 5-year-old children (i.e., before entering primary school) and their learning abilities during primary school [50,51]. Very few studies searched for the predictors of SLD in very early developmental stages [37].

In the current longitudinal retrospective study, we aimed to provide new and updated information on the prevalence of SLD in Italy. Moreover, we aimed to explore the variables that could contribute to predicting an SLD, focusing on (i) biological and environmental variables; (ii) the presence of a diagnosis of DLD when children were 4 to 5 years old; (iii) some communicative and linguistic measures collected when children were 27 to 30 months old, through the Italian version of MacArthur Bates Communicative Development Inventories, Words and Sentences (MB-CDI—WS—Short Form) [52].

We hypothesized to find a higher percentage of children with a diagnosis of SLD with respect to the previous studies conducted in Italy [9] due to the supposed increased sensitivity of the Italian school system’s teachers in identifying children with possible SLD and in inviting them to undertake a specific assessment in public health services or in other certified clinical centers specialized in specific learning disabilities. This increased sensitivity of teachers may partly be attributed to the three consensus conferences on SLD celebrated in Italy [3].

As for the contribution of biological and environmental variables, according to the literature, we hypothesized that the male sex/gender of children, familiarity with SLD, as well as low levels of parental education would result in increasing the probability of children receiving a diagnosis of SLD [12,16,17,18].

SLD is an umbrella term for various neurodevelopmental disorders such as dyslexia, dysgraphia, and dyscalculia; it may occur either in children with previous language delay and/or developmental language disorders or in children with typical language development. Thus, we hypothesized to find a weak relationship between measures from the early stages of language development and the presence of diagnosis of SLD [21,35,36,38,39].

## 2. Materials and Methods

### 2.1. Participants and Procedure

Participants were recruited through the permanent screening program carried out in Northwest Italy (province of Mantua), coordinated by the local health service, which was focused on the early identification of children at risk of LD. The screening program targeted families of 27- to 30-month-old children and the screening tool known as the Italian version of MacArthur Bates Communicative Development Inventories, Words and Sentences (MB-CDI—WS—Short Form) was used [52]. We accepted questionnaires regarding children up to 32 months of age.

The prevention unit of the Mantua local health service contacted the families of children by sending them home with a letter containing an informative brochure and an invitation to participate in a screening project presentation meeting. Parents who agreed to participate in the program signed an informed consent form and the local health service sent them the short form of the Italian MB-CDI to fill in.

**Figure 1:**
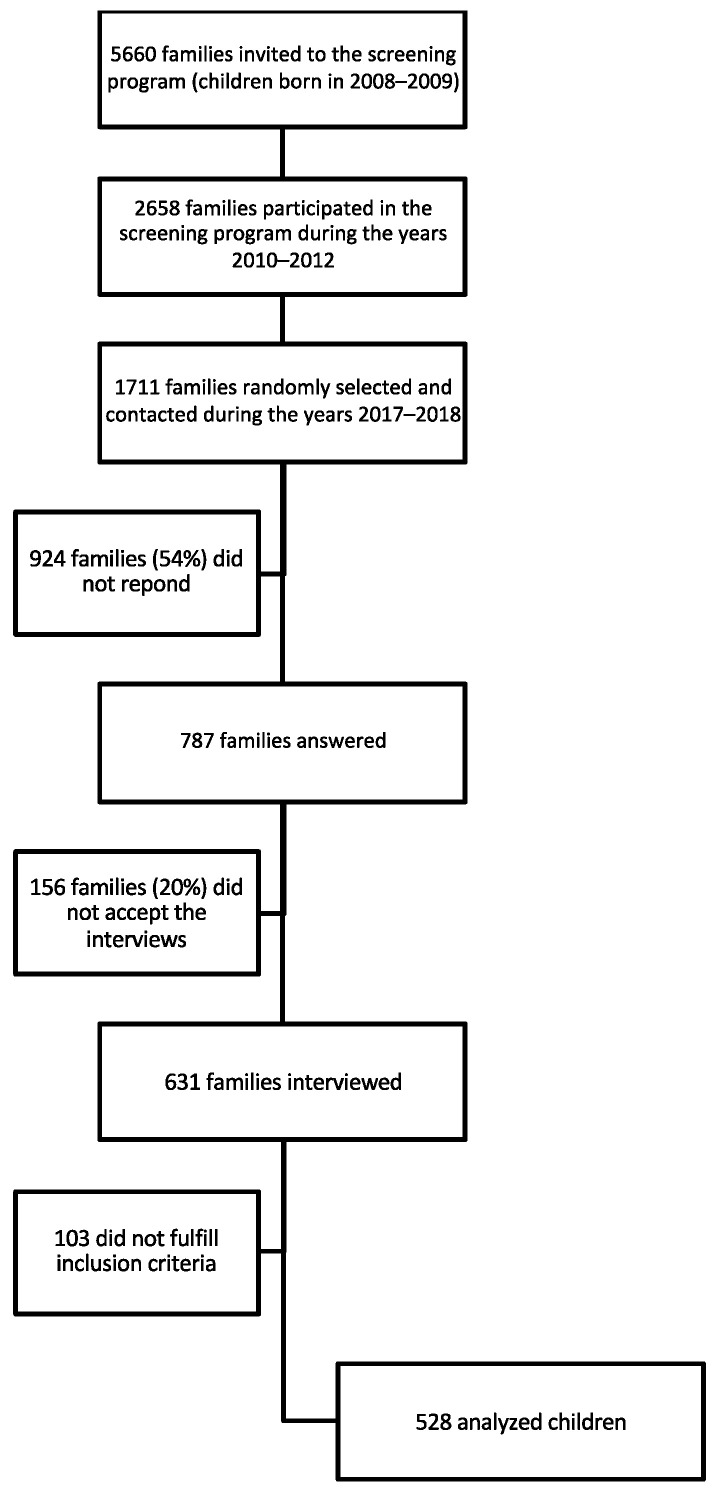
Flow chart of the recruitment.

As shown in Figure 1, during the first 3 years of the screening program (2010–2012), 5660 families of children born in the province of Mantua in 2008 (*n* = 2846) and in 2009 (*n* = 2814) were invited to participate in the screening program. Among these, 2658 families (47%) (*n* = 1195, 42% for children born in 2008; *n* = 1463, 52% for children born in 2009) agreed to participate in the screening program between 2010 and 2012. The overall attrition rate for the first 3 years of the screening program was 53%.

Among the 2658 families who participated in the screening program during the years 2010–2011, a sample of 1711 families (64% of the participating families) were randomly selected and contacted during the years 2017–2018, when children were enrolled in the primary school (about 6 years later), for a telephone interview. Among the 1711 families selected, 924 (54%) were found to be not contactable due to either unserviceable telephone numbers or a lack of response. The remaining 787 families (46%) answered our phone call and 631 of them (80%) accepted to provide us information about their child’s development and answered an interview (see below for details). The database was carefully checked and 103 datasets were excluded because children: (i) were older than 32 months at the screening step (*n* = 81); (ii) had received a diagnosis of neurodevelopmental disorder other than SLD (*n* = 12) (in order to focus on children whose learning disorder was specific and not associated with other neurodevelopmental disorders); (iii) were considered to be bilingual children because they were also exposed to a language different from Italian since birth by at least one of the parents, who in turn was a native speaker of that language (*n* = 10). The analyses were carried out on 528 children.

At the screening step, the mean observed age of children was 29.2 months (age range: 27–32 months); at the follow-up step, children were on average 8 years and 5 months old (age range: 7 years and 5 months to 9 years and 5 months). Participant recruitment and follow-up were approved by the Human Ethics Committee at the local health services and followed the principles outlined in the Declaration of Helsinki. Parents provided written informed consent to participate in the study.

### 2.2. Instruments

#### 2.2.1. MB-CDI Italian Words and Sentences Short Form

At the screening step, when children were 27 to 30 months old, the parents were asked to fill in the short form of the Italian Words and Sentences MB-CDI [52]. This tool was validated by 816 Italian children aged 18 to 36 months and showed a high concurrent validity with the Words and Sentences MB-CDI complete form [53], with the Words and Gestures MB-CDI short form [54] (r = 0.92 and r = 0.93, respectively), and with tools used to directly assess children’s language abilities [44,55].

It included four sections. The first section consisted of a list of 100 words. For each of these words, the parents had to mark those spontaneously produced by their child. The second section investigated the production of sentences. A single question asked whether the child had begun to combine words into sentences. Three response options were given: “Not Yet”, “Sometimes”, and “Often”. The third section investigated the level of complexity and morphosyntactic completeness of the sentences produced.

The fourth section investigated abilities related to language acquisition (e.g., verbal imitation, comprehension of decontextualized language, use of gestures, pretend play), using seven questions: (1) Does he/she use communicative gestures in order to name or to request? (2) Does he/she point to an object he/she wants? (3) Does he/she point to a picture or an object that he/she pays attention to in order to name it? (4) Does he/she pretend that one object is another object with a different function? (5) Does he/she understand when you speak about past and future events? (6) Does he/she repeat words just pronounced by an adult (three response options were given: “Not Yet”, “Sometimes”, and “Often”)? (7) How is the speech of the child (three response options were given: “His/her words are comprehended only by caregivers”, “He/she produces simplified words”, and “He/she speaks adult-like)? Additional information on the family’s educational status, the child’s medical history, and the family history for language and/or learning disorders was also collected. For more details, see Refs. [52,53,54].

#### 2.2.2. Parental Interview on Language and Academic Outcome

At the follow-up step, a structured parental interview was conducted to collect information about their language and academic development during the period from the two steps. The interview was developed ad hoc and included an introduction, 28 questions (divided into three sections), and an ending. Thirteen questions were yes/no questions, and the remaining fifteen questions required open answers.

The first section (8 questions) collected information about the child’s language development after the screening step. Parents were asked about the result of the screening program. Parents of children at risk of LD were asked whether the LD was confirmed by a neuropsychological assessment conducted in public health services or in other certified clinical centers; parents of children with typical language development were asked about whether eventual difficulties in communicative and linguistic development emerged later on. This section was very useful in familiarizing with the families and sharing a common ground when children were about 3 years old.

The second section (10 questions) collected information about eventual difficulties in communicative, linguistic, behavioral, and more general neuropsychological development when the children were 4 to 5 years old. Parents who reported difficulties in one or more of the aspects investigated were asked to specify whether the difficulties were confirmed during a neuropsychological assessment conducted in public health services or in other certified clinical centers, resulting in a certified diagnosis of DLD. Finally, they were asked to report whether their child underwent speech therapy or received any kind of support.

The third section (10 questions) collected information about eventual difficulties in academic abilities, as well as about eventual difficulties in behavior and attention during primary school. Parents who reported difficulties in one or more of the aspects investigated were asked to specify whether the difficulties were confirmed during a neuropsychological assessment conducted in public health services or in other certified clinical centers, resulting in a certified diagnosis of SLD. Finally, they were asked to report whether their child underwent speech therapy or received any kind of support.

Not all parents received all the questions. For example, parents who told us that their child did not show language difficulties at 4–5 years of age were not asked about an eventual diagnosis of DLD. To make the interview faster on the one hand and to minimize the risk of errors from the interviewer on the other, the schema of the interview was implemented on a Google module that automatically directed the subsequent question based on the previous answer provided by the parent. The duration of the interview was 5–10 min on average. The English translation of the parental interview is provided in Appendix A (Table A1).

### 2.3. Coding and Measures

Starting with evidence from the literature on the possible predictors of SLD, as reported in the Introduction, the following biological, environmental, communicative, and linguistic variables were coded and considered in the present study (Table 1).

In addition, in the second step, when analyzing the data of the parental interviews, we coded two additional variables: (i) diagnosis of developmental language disorder (0 = absence; 1 = presence) and (ii) diagnosis of specific learning disorder (0 = absence; 1 = presence).

Only children whose parents reported that they (i) received a diagnosis of DLD and/or SLD following the above reported diagnostic procedure (i.e., after receiving a neuropsychological assessment conducted in public health services or in other certified clinical centers, resulting in a certified diagnosis) and (ii) were offered care for DLD and/or SLD, were considered to have a DLD and/or an SLD.

#### Statistical Analysis

As for the data about the prevalence of SLD in Italy, the Chi squared test was applied to verify the association among the diagnoses of SLD, the diagnoses of DLD, and the presence of LD expressed in terms of absolute frequency (*n*) and percentage (%).

Since this study mainly focused on early communicative and linguistic measures, the multivariable logistic regression models and the stepwise selection methods could suggest which of the measures considered here mostly contributed to the early identification of a risk of SLD. In order to identify independent predictors of SLD, DLD, and vocabulary size, logistic regression models were applied to identify which independent variables were significantly associated with the dependent one, considering the following: child’s sex, familial risk of language and/or learning disorders, mother’s level of education, use of communicative gestures to name or to request, requesting pointing, declarative pointing, verbal imitation frequency, decontextualized comprehension, phonological accuracy, and word–word combination use. In addition, for predicting the probability of diagnosis of DLD, vocabulary size was added as an independent variable and, for predicting the probability of SLD, the presence of DLD and vocabulary size were added as independent variables.

In order to individuate the best multivariable model for each dependent variable, the stepwise selection methods were applied, considering only the variables with a *p* value < 0.10 at the univariable analysis. The stepwise procedure selected, among these, the independent variables to be included (or not) in the final model. The results of the models were presented in terms of Odds Ratio (OR), reporting the corresponding 95% Confidence Interval (95% CI). The *p* values of each final model were corrected for multiple testing using the Benjamini–Hochberg method [56]. The accuracy of each model was quantified by the Area Under Curve (AUC) of the individuated multivariable model. All data were analyzed using R (version 4.0.2, R Foundation for Statistical Computing, Vienna, Austria). A *p*-value of <0.05 was considered statistically significant.

## 3. Results

As shown in Table 2, from the 528 interviews, it emerged that 37 children (7.01%) had received a diagnosis of SLD, whereas the remaining 491 (92.99%) had not, thus they could be considered as children with typical learning development. 

About one third of the children with SLD (24 children; 64.86%) had not received a diagnosis of DLD nor were they found to have LD, thus they could be considered as children with typical language development; of the remaining, 11 children (29.73%) had received a diagnosis of DLD (2 of them without LD, the remaining 9 with previous LD), whereas the last 2 (4.41%) had a history of LD but had not received a diagnosis of DLD.

More than three-quarters of the children without SLD (391; 79.63%), and thus with typical learning development, had not received a diagnosis of DLD nor were they found to have LD. Of the remaining, 50 children (10.18%) had received a diagnosis of DLD (27 of them without LD, the remaining 23 with previous LD) and 50 (10.18%) had a history of LD but had not received a diagnosis of DLD. 

The differences in the distribution of these data were statistically significant (Chi-squared (3) = 23.65; *p* < 0.001). In particular, the percentage of children with DLD with previous LD was higher among those with SLD compared with those without SLD.

As shown in Table 3, univariable logistic analyses with SLD as the dependent variable showed that child’s sex/gender, verbal imitation frequency, vocabulary size, word–word combination, and presence of DLD were statistically significant. Pretend play and decontextualized comprehension were not entered in the models, since no child who did not use pretend play and no child who did not show decontextualized comprehension presented SLD, thus the model was not able to estimate the contribution of these two variables. The probability of receiving a diagnosis of SLD was higher for boys (2.74 times higher than for girls), for children who, at 27–30 months of age, did not imitate words (4.27 times higher than for children who imitated words), had a vocabulary size below the 10th percentile (2.57 times higher than for children with a vocabulary size above the 10th percentile), did not yet combine words in sentences (2.33 times higher than for children who produced word–word combinations), and for children who received a diagnosis of DLD when they were 4–5 years old (3.77 times higher than for children without a diagnosis of DLD). The best multivariable model, which included child’s sex/gender, family history of SLD, and presence of DLD, had a moderate accuracy in individuating children with SLD (AUC = 0.71; 95% CI = 0.63–0.80). See Table 3 for details of the univariable analyses and of the best multivariable model.

**Figure 2:**
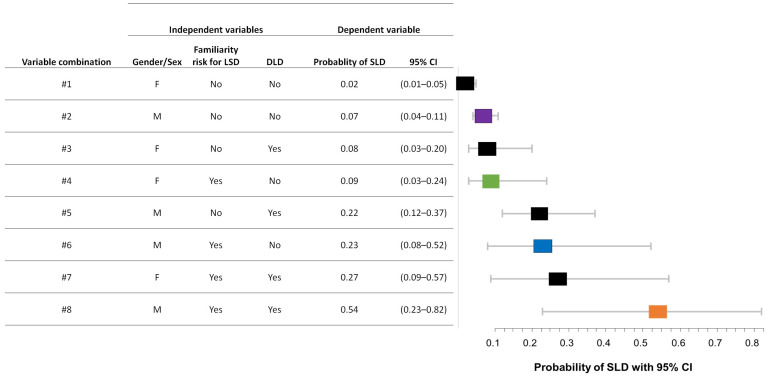
Probability to receive a diagnosis of SLD associated with the possible combinations among the different levels of the variables entered in the best multivariable model.

On the basis of the multivariable model, we estimated the probability to receive a diagnosis of SLD according to the different levels of the variables included in the model. As shown in Figure 2, girls with no familial risk of SLD and without a diagnosis of DLD had the lowest probability of receiving a diagnosis of SLD (2%), while girls with a familial risk of SLD with a diagnosis of DLD had the highest probability of receiving a diagnosis of SLD (54%). The probabilities associated with the possible combinations among the different levels of the variables entered in the best multivariable model are shown in Figure 2.

As shown in Table 4, univariable logistic analyses with DLD as the dependent variable showed that pretend play, verbal imitation frequency, vocabulary size, and word–word combination were statistically significant. Requesting pointing and use of communicative gestures were not entered in the models, since no child who did not use requesting pointing and no child who did not use communicative gestures received a diagnosis of DLD, thus the model was not able to estimate the contribution of these two variables.The probability of receiving a diagnosis of DLD was higher for children who, at 27–30 months of age, did not use pretend play (2.63 times higher than for children who used pretend play), did not imitate words (11.2 times higher than for children who imitated words), had a vocabulary size below the 10th percentile (7.53 times higher than for children with a vocabulary size above the 10th percentile), and did not combine words in sentences (6.77 times higher than for children who produced word–word combinations). The best multivariable model, which included verbal imitation frequency and vocabulary size, had a moderate accuracy in individuating children with DLD (AUC = 0.69; 95% CI = 0.62–0.76). See Table 4 for details of the univariable analyses and the best multivariable model.

**Figure 3:**
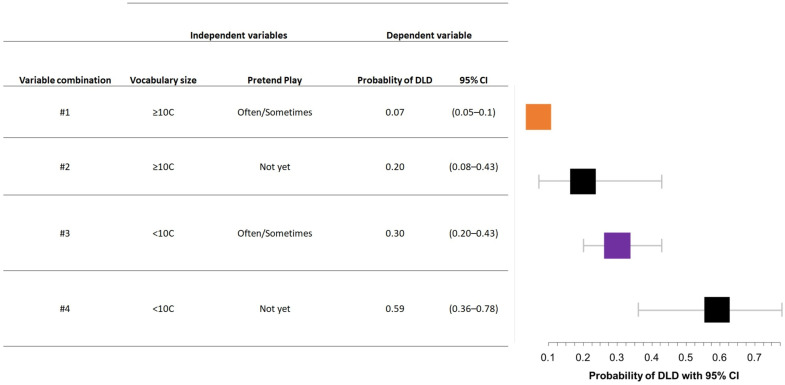
Probability of receiving a diagnosis of DLD associated with the possible combinations among the different levels of the variables entered in the best multivariable model.

On the basis of the multivariable model, we estimated the probability of receiving a diagnosis of DLD according to the different levels of the variables included in the model. As shown in Figure 3, children with a vocabulary size above the 10th percentile and who showed pretend play at 27–30 months of age had the lowest probability of receiving a diagnosis of DLD at 4–5 years of age (7%), while children with a vocabulary size below the 10th percentile and who did not yet use pretend play at 27–30 months of age had the highest probability of receiving a diagnosis of DLD when they were 4–5-year-old (59%). The probabilities associated with the possible combinations among the different levels of the variables entered in the best multivariable model are shown in Figure 3.

As shown in Table 5, univariable logistic analyses with vocabulary size as the dependent variable showed that children’s sex/gender, mother’s level of education, decontextualized comprehension, and verbal imitation frequency were statistically significant. Requesting pointing and declarative pointing were not entered in the models, since no child who did not use requesting pointing and no child who did not use declarative pointing had a vocabulary size below the 10th percentile, thus the model was not able to estimate the contribution of these two variables.The probability of having a vocabulary size below the 10th percentile at 27–30 months of age was higher for children who were males (1.67 times higher than for girls), had mothers with a primary/secondary level of education (2.33 times higher than for children with mothers with a university degree), did not yet show decontextualized comprehension (11.25 times higher than for children who showed decontextualized comprehension), and did not yet imitate words (44.43 times higher than for children who imitated words). The best multivariable model, which included decontextualized comprehension and verbal imitation frequency, had a moderate accuracy when individuating children with a vocabulary size below the 10th percentile (AUC = 0.63; 95% CI = 0.58–0.68). See Table 5 for details of the univariable analyses and the best multivariable model.

**Figure 4:**
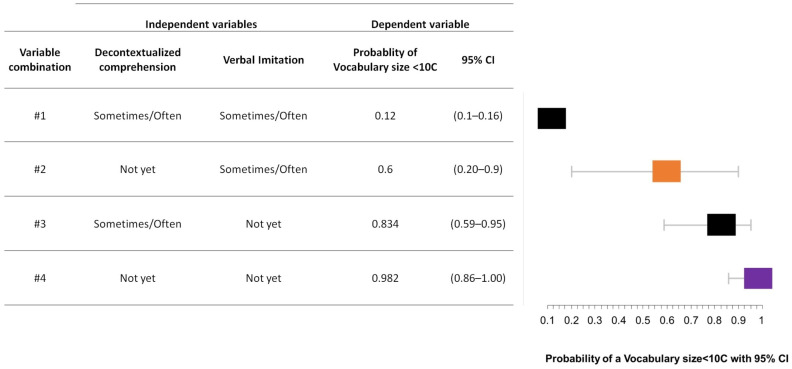
Probability of having a vocabulary size below the 10th percentile at 27–30 months of age associated with the possible combinations among the different levels of the variables entered in the best multivariable model.

On the basis of the multivariable model, we estimated the probability of having a vocabulary size below the 10th percentile at 27–30 months of age according to the different levels of the variables included in the model. As shown in Figure 4, children who showed decontextualized comprehension and used verbal imitation had the lowest probability of having a vocabulary size below the 10th percentile (12%), while children who did not show decontextualized comprehension and who did not use verbal imitation had the highest probability of having a vocabulary size below the 10th percentile (98%). The probabilities associated with the possible combinations among the different levels of the variables entered in the best multivariable model are shown in Figure 4.

## 4. Discussion

In the current longitudinal retrospective study, we aimed to provided new data on the prevalence of SLD diagnoses in Italian children attending primary school and to explore whether it could be possible to predict an SLD starting from distal predictors, collected when children were 27–30 months old (age at which LD can be detected), from proximal predictors, collected when children were 4–5 years old (age at which a DLD can be diagnosed), as well as from biological and environmental variables.

From the 528 families who participated in both steps of the study (screening for LD and parental interview) and who were included in the analyses, it emerged that 7.01% of the children of our sample had received a diagnosis of SLD. The prevalence we found was in accordance with the prevalence found in other studies, as reported in the very recent systematic review and meta-analysis conducted by Yang and colleagues, particularly with the prevalence reported by studies that had similar characteristics to the one reported here (i.e., those conducted in Europe), with random sampling and with a sample size of 500–1000 participants [5]. In contrast, the prevalence we found was substantially higher than that reported in a recent Italian study [9]. The difference from the Barbiero and colleagues’ study, notwithstanding the fact that both studies were conducted in Italy, could be partly explained by the different sample sizes (*n* = 528 in the current study; *n* ≈ 10,000 in [9]), as well as by the different periods in which the studies were conducted. The prevalence of SLD diagnoses in the current study referred to the years 2017–2018, whereas the prevalence of the other study referred to the years 2008–2013. The increase in the percentage of children with a diagnosis of SLD could also be due to the progressive growth of the Italian school system’s sensitivity and ability to correctly identify children to be sent for certification.

About two-thirds of children with an SLD (64.86%) had typical language development because they did not have a history of previous DLD nor were they found to have LD. About 30% of children with an SLD had a history of DLD, whereas 5% had a history of LD but had not received a diagnosis of DLD. This wide variability in the outcome does not help in understanding whether DLD and SLD are distinct disorders without overlaps or whether they are characterized by strong associations and continuity between them, as suggested in other studies (e.g., [24]).

Data collected in the current study relied on parental interviews and thus we were unaware of neuropsychological profile(s) as well as phenotypic features of children who had received a diagnosis of either SLD or DLD. Furthermore, in light of previous results, we hypothesized that children with disorders that had different etiologies and were characterized by different phenotypic features fell under the same diagnostic label (i.e., DLD); some of them supported the idea of a continuum between DLD and SLD and some did not.

The same was true for the diagnosis of SLD; within this classification, we found children characterized by different neuropsychological profiles, some of them with a previous diagnosis of DLD and some without. For example, in our study, among children with typical learning development (i.e., children without an SLD), about 10% had received a diagnosis of DLD and about 10% had a history of LD but had not received a diagnosis of DLD. These data were in accordance with the multiple deficit model for dyslexia, proposed by refusing the existence of single core deficits, as well as with recent theoretical perspectives that offer evidence for the multifactorial and multidimensional nature of neurodevelopmental disorders, including SLD (for dyslexia see, e.g., [57]).

Looking at biological, environmental, and individual proximal and distal measures able to predict a diagnosis of SLD, we found that sex/gender, verbal imitation, vocabulary size, word–word combination, and the presence of DLD significantly increased the probability of receiving a diagnosis of SLD. This result confirmed that poor language skills and/or the presence of DLD must be considered as risk factors of SLD [22,23,25,58]. In addition, the multivariable model, which selected the mix of variables that better explained the presence of the independent one, called into question children’s sex/gender, familial risk, and the presence of DLD. Indeed, being a boy with a familial risk of SLD and with a diagnosis of DLD increased the probability of receiving a diagnosis of SLD (54%). This result was in accordance with studies that found that the sex/gender of children and the familial risk of DLD or SLD, as well as serious weaknesses in language development, were significantly associated with the presence of SLD [19,20,21,22,23].

The data and information we collected over a 6-year period also allowed us to investigate possible predictors of the diagnosis of DLD. We found that the probability of receiving a diagnosis of DLD was higher for children who, at 27–30 months of age, either did not use pretend play, did not imitate words, had a vocabulary size below the 10th percentile, or did not combine words in sentences. A vocabulary size below the 10th percentile and the lack of word–word combination at 24–30 months of age were the criteria for defining children as late talkers. From our data, in particular from the results of the multivariable analysis, it emerged that children with the highest probability (59%) of receiving a diagnosis of DLD were those who had a vocabulary size below the 10th percentile and who did not imitate words. These results confirmed a continuum between LD and DLD on the one hand and the wide variability in the outcome of children with LD, some of them spontaneously recovering their gap and others resulting in DLD, on the other hand [43].

As for vocabulary size at 27–30 months of age, univariable analyses showed that the probability of receiving a diagnosis of DLD was higher for children who were a boy, had a mother with a low level of education, did not show decontextualized comprehension, or did not use verbal imitation. These results were in accordance with research that considered male sex/gender and familial risk as risk factors of a language delay (i.e., vocabulary size below the 10th percentile at 24–30 months of age) [14,15,17,38]. In addition, from the multivariable analysis, it emerged that children with the highest probability (98%) of having a language delay were those who did not show decontextualized comprehension and did not imitate words. With respect to the weaknesses in decontextualized comprehension and verbal imitation, they had already been found to be associated with vocabulary size in a sample of 26- to 35-month-old Italian late-talking children [44].

Longitudinal studies starting from early childhood, aimed at defining the developmental trajectories of school-age children who will show SLD, are still few and suffer from the high complexity of the topic. It has become necessary for future studies to consider, identify, and distinguish distal from proximal predictors. As distal predictors, general mechanisms of processing (even at a very early developmental stage) that may contribute to the later emergence of SLD should also be considered. As proximal predictors, measured in the later preschool years, which are temporally closer to the acquisition of a given skill, domain-specific processes (pertaining to reading and writing) should also be measured. It seems obvious that the weight of predictors becomes stronger as we become temporally closer to the clinical manifestation of the disorder and within the specific cognitive domain. Thus, measures collected in later preschool years appear to better predict SLD.

However, we believe it is useful to identify, from a developmental perspective, very early predictive indices of SLD (while fully understanding that they will be able to explain significant but small portions of the variance) in order to properly identify children who can benefit from eventual interventions at early developmental stages.

By synthesizing the results of this research, it emerged that boys with a familial risk of SLD, who had already received a DLD diagnosis, had a 54% probability of having an SLD diagnosis. In turn, children with language delay and who did not imitate words at 27–30 months of age had a 59% probability of having a diagnosis of DLD. In turn, children who did not show decontextualized comprehension and who did not imitate words had a 98% probability of having a language delay.

## 5. Limitations of the Current Study

This study had several limitations that should be taken into account in interpreting the results reported. First of all, we did not administer any direct assessment of the children. The study was based on the results of an MB-CDI parental questionnaire when the children were 27–30 months old and on a detailed interview with one parent when the children were in primary school. We did not ask parents for scores in specific tests, nor did we ask them to report on generic linguistic and/or learning difficulties of their child at school age. However, in an attempt to mitigate the impact of this limitation, we asked parents, with specific questions and related sub-questions, to report information about eventual diagnoses of DLD and SLD made by specific professionals working in public health services or in other certified clinical centers after a direct neuropsychological assessment with standardized tests. Only children that received a DLD and/or an SLD diagnosis (as reported by parents) following the above criteria were considered to have a DLD and/or an SLD. In addition, another limitation to be highlighted refers to the interview we used in this study. The interview was developed ad hoc for the current study, thus further studies are necessary in order to investigate its validity and reliability, considering data obtained by a direct evaluation of children. For the same reasons, we decided to not ask parents to specify the type of SLD (i.e., dyslexia, dyscalculia, or dysgraphia), thus considering the more comprehensive diagnosis of SLD.

## 6. Conclusions

We provided new and updated data on the prevalence of SLD diagnoses in Italian children attending primary school. From this study, it emerged that the prevalence of children with SLD in Italy was 7.01%. We identified biological, environmental, and children’s behavioral variables that could be viewed as risk factors of a diagnosis of SLD, of DLD, and to be a late talker, as well as patterns of these variables that increased the probability of a given child to present SLD, DLD, or language delay. These results could help clinicians by guiding them in assessing composite factors, taking into consideration those factors that add the risk of having certain language and/or learning disabilities.

## Figures and Tables

**Table 1:** Domains and variables coded in the first step of the study.

Domain	Variable	Coding
Biological factor (data taken from the anamnestic section of the MB-CDI)	child’s sex/gender	0 = girl;
		1 = boy
	family history of language and/or learning disorders as a proxy of familial risk of SLD	0 = absence;
		1 = presence
Environmental factor (data taken from the anamnestic section of the MB-CDI)	mother’s level of education	0 = primary/secondary school;
		1 = high school;
		2 = university
Communicative and linguistic abilities (data taken from the MB-CDI)	use communicative gestures	0 = not yet;
	to name or to request	1 = sometimes/often;
	requesting pointing	0 = not yet;
		1 = sometimes/often
	declarative pointing	0 = not yet;
		1 = sometimes/often
	verbal imitation frequency	0 = not yet;
		1 = sometimes/often
	pretending play	0 = not yet;
		1 = sometimes/often
	decontextualized	0 = not yet;
	comprehension	1 = sometimes/often
	phonological accuracy	0 = only caregivers understand him/her;
		1 = simplifies words;
		2 = speaks like an adult
	vocabulary size	0 = <10th percentile;
		1 = >10th percentile
	word–word combination use	0 = not yet;
		1 = sometimes/often

**Table 2:** Number and percentage of children with and without SLD who had either typical (no previous LD or DLD) or atypical (LD, DLD, or both) language development.

	No Previous LD or DLD	Previous LD (without DLD)	DLD (without LD)	DLD (with Previous LD)
Children with SLD *n* = 37	24 (64.86%)	2 (5.41%)	2 (5.41%)	9 (24.32%)
Children without SLD *n* = 491	391 (79.63%)	50 (10.18%)	27 (5.5%)	23 (4.68%)

**Table 3:** Details of the univariable analyses and of the best multivariable model with diagnosis of SLD as the dependent variable.

		Dependent Variable: Presence of SLD					
		Univariable Analysis			Multivariable Analysis		
Independent Variable	Category	OR	95% CI	*p* Value	OR	95% CI	Adj *p* Value
Child’s sex/gender	M vs. F	2.74	1.25–6.67	0.016	3.33	1.28–7.69	0.017
Familial risk of LSD	Yes vs. No	3.04	0.84–8.72	0.056	4.12	1.19–14.29	0.028
Mother’s level of education	Primary/secondary vs. High school	2.0	0.87–4.54	0.103			
	Primary/secondary vs. University	2.50	0.88–7.14	0.086	Not selected by stepwise		
Use of communicative gestures	Not yet vs. Sometimes/Often	1.89	0.41–9.09	0.411			
Requesting pointing	Not yet vs. Sometimes/Often	1.85	0.22–16.67	0.568			
Declarative pointing	Not yet vs. Sometimes/Often	0.84	0.32–2.22	0.722			
Verbal imitation	Not yet vs. Sometimes/Often	4.27	1.48–12.33	0.007	Not selected by stepwise		
Phonological accuracy	Only caregivers understand him/her vs. simplifies words/speaks like an adult	1.12	0.54–2.32	0.771			
Vocabulary size	<10th percentile vs. >10th percentile	2.57	1.12–5.55	0.019	Not selected by stepwise		
Word–word combination	Not yet vs. Sometimes/Often	2.33	1.04–5.17	0.038	Not selected by stepwise		
Presence of DLD	Yes vs. No	3.77	1.62–8.26	0.001	3.88	1.69–8.91	0.002

Adj *p* value = *p* value corrected for multiple testing.

**Table 4:** Details of the univariable analyses and the best multivariable model with diagnosis of DLD as the dependent variable.

		Dependent Variable: Presence of DLD					
		Univariable Analysis			Multivariable Analysis		
Independent Variable	Category	OR	95% CI	*p* Value	OR	95% CI	Adj *p* Value
Child’s sex/gender	M vs. F	1.22	0.70–2.17	0.482			
Familial risk of LSD	Yes vs. No	2.08	0.67–5.44	0.163			
Mother’s level of education	Primary/secondary vs. High school	1.25	0.58–2.63	0.57			
	Primary/secondary vs. University	1.22	0.52–2.86	0.653			
Declarative pointing	Not yet vs. Sometimes/Often	0.56	0.23–1.35	0.195			
Pretend play	Not yet vs. Sometimes/Often	2.63	1.13–6.15	0.025	Not selected by stepwise		
Decontextualized comprehension	Not yet vs. Sometimes/Often	1.55	0.18–13.52	0.69			
Verbal imitation	Not yet vs. Sometimes/Often	11.2	4.61–27.35	<0.001	3.22	1.14–9.09	0.028
Phonological accuracy	Only caregivers understand him/her vs. simplifies words/speaks like an adult	1.45	0.83–2.55	0.197			
Vocabulary size	<10th percentile vs. >10th percentile	7.53	4.09–13.9	<0.001	5.73	2.95–11.14	<0.001
Word–word combination	Not yet vs. Sometimes/Often	6.77	3.71–12.32	<0.001	Not selected by stepwise		

Adj *p* value = *p* value corrected for multiple testing.

**Table 5:** Details of the univariable analyses and the best multivariable model, with vocabulary size as the dependent variable.

		Univariable Analysis			Multivariable Analysis		
Independent Variable	Category	OR	95% CI	*p* Value	OR	95% CI	Adj *p* Value
Child’s sex/gender	M vs. F	1.67	1.03–2.70	0.037	Not selected by stepwise		
Familial risk of SLD	Yes vs. No	1.72	0.67–4.44	0.262			
Mother’s level of education	Primary/secondary vs. High school	1.56	0.83–2.86	0.163	Not selected by stepwise		
	Primary/secondary vs. University	2.33	1.09–5.00	0.03			
Use of communicative gestures	Not yet vs. Sometimes/Often	0.99	0.52–1.89	0.982			
Pretend play	Not yet vs. Sometimes/Often	2.15	0.96–4.81	0.063	Not selected by stepwise		
Decontextualized comprehension	Not yet vs. Sometimes/Often	11.25	2.03–62.5	0.006	11.11	1.78–50	0.017
Verbal imitation	Not yet vs. Sometimes/Often	44.43	12.8–154.53	<0.001	33.33	10–100	<0.001
Phonological accuracy	Only caregivers understand him/her vs. simplifies words/speaks like an adult	1.23	0.49–1.34	0.416			

Adj *p* value = *p* value corrected for multiple testing.

## Data Availability

The datasets generated and/or analyzed during the current study are available from the corresponding author on reasonable request.

## Appendix A

**Table A1:** English translation of the parental interview.

Question Number	Question	Answer
Section 1		
Introduction	As you may recall, when your child was about three years old you participated in a screening program by the Mantua Local Health Authority to check whether your child’s communication and language development was adequate for his or her age. On that occasion, you were asked to fill in a questionnaire where you had to indicate how many words he said and whether he produced sentences. Do you remember? With this interview, we want to find out how your child’s language has developed since that point. The information you give us, as it is sensitive information, will not be disseminated outside the research and will be kept anonymous. The interview will take about 5 min.	
1	After completing the questionnaire, were you contacted by a pediatrician and/or local health services about a language delay in your child?	○ Yes ○ No
2	Did your child have a follow-up within a few months after that contact at the local health services (LHS) or at a specialized clinical center?	○ Yes, at LHS ○ Yes, at another clinical center ○ No
3	Did the follow-up visit confirm the outcome of the screening, i.e., the result of the questionnaire?	○ Yes ○ No
4	Did the follow-up visit reveal any developmental difficulties other than language difficulty?	○ Yes ○ No
5	What kind of difficulties?	text
6	Has the child, as a consequence of the difficulties that were noted, undergone therapy?	○ Yes ○ No
7	Who has been following the child’s development?	○ Child neuropsychiatrist ○ Speech-language pathologist ○ Psychologist ○ Other
8	For how long?	text
Section 2		
9	Around the age of 4–5, did you notice any language difficulties in the child?	○ Yes ○ No
10	What kind of difficulties?	text
11	Have other developmental and/or behavioral difficulties arisen?	○ Yes ○ No
12	What kind of difficulties?	text
13	Was a clinical evaluation carried out after the difficulties emerged?	○ Yes ○ No
14	Who performed this evaluation?	○ Child neuropsychiatrist ○ Speech-language pathologist ○ Psychologist ○ Other
15	What diagnosis was given in the clinician’s report?	text
16	Has the child, as a result of the difficulties that arose, followed or continued to follow therapy?	○ Yes ○ No
17	Who oversaw that therapy?	○ Child neuropsychiatrist ○ Speech-language pathologist ○ Psychologist ○ Other
18	For how long?	text
Section 3		
19	Did any difficulties in reading and/or in writing emerge in the first two years of primary school that caused concern to teachers?	○ Yes ○ No
20	What difficulties caused concern?	text
21	Have other difficulties (e.g., in other subjects, behavior, attention) been reported?	○ Yes ○ No
22	What kind of difficulties?	text
23	Was a clinical evaluation carried out after the difficulties emerged?	○ Yes ○ No
24	Who performed the evaluation?	○ Child neuropsychiatrist ○ Speech-language pathologist ○ Psychologist ○ Other
25	What diagnosis was given in the clinician’s report?	text
26	Has the child, as a result of the difficulties that arose, followed or continued to follow therapy?	○ Yes ○ No
27	Who followed and assessed the child’s progress?	○ Child neuropsychiatrist ○ Speech-language pathologist ○ Psychologist ○ Other
28	For how long?	text
Ending	If you want to tell us any comments or considerations you have regarding the child’s developmental supervision and success or lack of it, you are welcome. Thank you.	text
